# Nanocarbon-Coated Porous Anodic Alumina for Bionic Devices

**DOI:** 10.3390/ma8084992

**Published:** 2015-08-05

**Authors:** Morteza Aramesh, Wei Tong, Kate Fox, Ann Turnley, Dong Han Seo, Steven Prawer, Kostya (Ken) Ostrikov

**Affiliations:** 1 School of Physics, the University of Melbourne, Melbourne, VIC 3010, Australia; E-Mails: w.tong4@student.unimelb.edu.au (W.T.); s.prawer@unimelb.edu.au (S.P.); 2 School of Chemistry, Physics, and Mechanical Engineering, Queensland University of Technology, Brisbane, QLD 4000, Australia; 3 Plasma Nanoscience Laboratories, Commonwealth Scientific and Industrial Research Organisation (CSIRO), PO Box 218, Lindfield, NSW 2070, Australia; E-Mail: Michael.Seo@csiro.au; 4 Center for Additive Manufacturing, School of Aerospace, Mechanical and Manufacturing Engineering, RMIT University, Carlton, VIC 3053, Australia; E-Mail: kfox@unimelb.edu.au; 5 Department of Anatomy and Neuroscience, the University of Melbourne, Parkville, VIC 3010, Australia; E-Mail: turnley@unimelb.edu.au

**Keywords:** nanocarbon coating, nanoporous aluminum oxide, diamond-like carbon, chemical resistivity, neural compatibility, bionic devices

## Abstract

A highly-stable and biocompatible nanoporous electrode is demonstrated herein. The electrode is based on a porous anodic alumina which is conformally coated with an ultra-thin layer of diamond-like carbon. The nanocarbon coating plays an essential role for the chemical stability and biocompatibility of the electrodes; thus, the coated electrodes are ideally suited for biomedical applications. The corrosion resistance of the proposed electrodes was tested under extreme chemical conditions, such as in boiling acidic/alkali environments. The nanostructured morphology and the surface chemistry of the electrodes were maintained after wet/dry chemical corrosion tests. The non-cytotoxicity of the electrodes was tested by standard toxicity tests using mouse fibroblasts and cortical neurons. Furthermore, the cell–electrode interaction of cortical neurons with nanocarbon coated nanoporous anodic alumina was studied *in vitro*. Cortical neurons were found to attach and spread to the nanocarbon coated electrodes without using additional biomolecules, whilst no cell attachment was observed on the surface of the bare anodic alumina. Neurite growth appeared to be sensitive to nanotopographical features of the electrodes. The proposed electrodes show a great promise for practical applications such as retinal prostheses and bionic implants in general.

## 1. Introduction

Diamond and diamond-like carbon (DLC) are attractive candidate materials for implantation into the body due to their excellent properties, such as high chemical stability, superb biocompatibility and tunable surface chemistry [1,2,3,4,5,6]. Diamond related materials are not only used for cell modulation (such as osteoblasts [7], fibroblasts [8], cortical neurons [9], and cortical stem cells [10]), but they also show great promise for biosensing platforms [11,12,13,14,15,16]. Diamond related materials including DLC are used in high-end biomedical applications such as implantable orthopaedic devices, dental and cardiovascular implants and bionic devices [2,17,18,19]. DLC coatings in orthopedic applications reduce wear, corrosion, debris formation and thrombogenicity by minimizing the platelet adhesion and activation [3].

As another example, diamond based retinal prosthesis (as part of the Bionic Vision Australia’s Bionic Eye Program) have shown great promise for the restoration of sight to blind patients who suffer from retinal diseases such as retinitis pigmentosa [9,19,20,21]. The implanted bionic diamond device has an array of neurally-interfaced stimulating microelectrodes which can communicate with cerebral cortex. These brain-computer interfaces (BCIs), can link the brain (through neurons) to the external world by means of computer processing [22,23]. The performance of these devices critically depends on the tissue-electrode interfaces (*i.e.*, neural compatibility with the electrodes).

Although diamond/DLC coatings can improve the performance of a device, the high level of stress and poor adhesion in aqueous environment can result in instability of the coatings [4]. Therefore, any coating technique applied should be carefully considered.

Additionally, applications of three-dimensional diamond and DLC nanostructures have been hindered due to the difficult processing of these materials. So far, most of the proposed prosthetic implants are based on planar devices [24]. However, it has been shown that three-dimensional structures—such as scaffolds and nanoporous materials—can significantly influence the tissue-device interface (such as neural compatibility) by improving the cell adhesion, migration, and proliferation [25,26,27,28]. The three-dimensional nanoporous structures are particularly interesting since they mimic the three-dimensional nature of tissue in organisms [29,30,31,32]. The nano-featured scaffolds have the potential to be used for cell isolation, nerve repair, tissue engineering after injury or disease, neural probes, neural prosthetics and brain–computer interfaces for recording and stimulating neurons *in vivo*, *in vitro* neuron-based sensors and neuronal networks [28].

**Figure 1:**
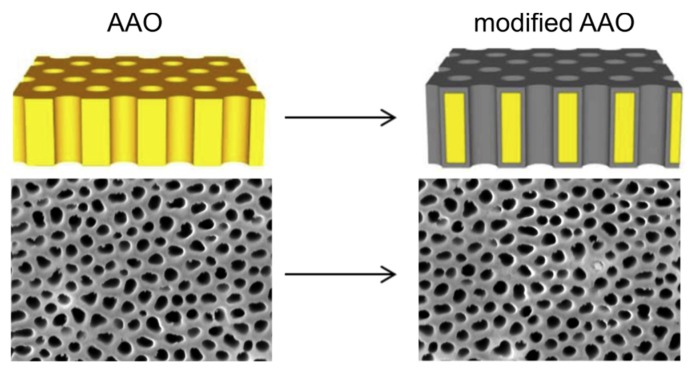
The entire surface of nanoporous anodic alumina (AAO) is coated with an ultrathin diamond-like carbon layer.

Recently, we have reported on a facile fabrication technique to produce three-dimensional nano-carbon structures with properties comparable with diamond [33] (Figure 1). We have successfully coated the entire surface of nanoporous anodic alumina (AAO) with diamond-like carbon (the detailed fabrication technique can be found elsewhere [33]). AAO is a nanoporous structure with tunable chemical and physical properties, which can be easily fabricated at a large scale with a straight-forward electrochemical process [34]. However, practical biomedical applications of AAO are scarce due to poor (bio-)chemical stability of the oxide [35]. The conformal coating of AAO with an ultra-thin DLC layer greatly enhances its chemical stability and non-cytotoxicity [14,33]. The chemically stable and non-cytotoxicity nanoporous DLC-AAO provides unique features for range of biomedical and biological applications such as bionic devices, 3D scaffolds, membrane for cell growth and nerve repair [35].

Here, we provide a brief summary of the properties of the coated materials, and present the effect on neural growth on the fabricated membranes. The results suggest that DLC-AAO with its three-dimensional nanocarbon structure has a great promise for applications in bionic electrodes and 3D cell culture.

## 2. Results and Discussion

### 2.1. Conductivity of the Electrodes

The conductivity of a carbon coated AAO electrode varies from 10 to 200 kΩ, depending on the atomic structure of the carbon layer. Basically, the ratio of the sp^2^/sp^3^ bonded carbon atoms determines the conductivity of the layer (*i.e.*, higher ratio of sp^2^ bonded carbon results in higher conductivity). The atomic structure of the carbon layer can be controlled during the deposition process by changing the plasma parameters such as temperature. We have previously shown that higher temperature results in formation of carbon layer with higher sp^2^ content [33]. A common DLC-AAO electrode (with reported plasma parameters in this paper) has a conductivity of ~200 kΩ. However, the detailed analysis of conductivity effects on the biocompatibility of the electrodes is out of the scope of this paper and will be the subject of further studies.

### 2.2. Chemical Stability

Long-term stability is a crucial factor for an implantable device. *In vivo* applications especially require materials that exhibit excellent stability in the biological environment. Some studies have demonstrated that AAO (or modified AAO) can be stable under moderate physiological conditions and presents negligible cytotoxicity [35,36,37,38,39,40]. However, for some bio-device processing purposes (e.g., sterilization or functionalization) it is desirable to have a material which can tolerate slightly stronger acid or basic environments with minimum structural damages. The sp^3^-bonded carbon materials, on the other hand, are well-known for their chemical resistance.

**Figure 2:**
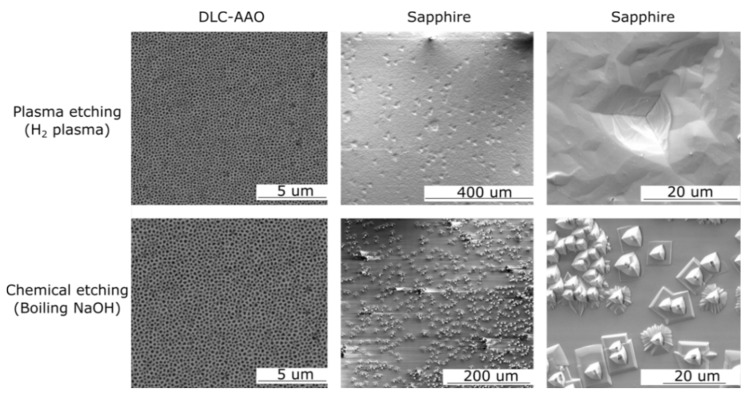
DLC-AAO and sapphire after wet/dry chemical etching. DLC-AAO’s structure did not change after etching, while sapphire’s surface was significantly damaged.

Table 1 shows the results of chemical resistance of AAO and DLC-AAO films in comparison to diamond and sapphire. DLC-AAO demonstrated excellent corrosion resistance against all tested chemicals with no signs of degradation, similar to diamond, whereas AAO was completely etched in those acidic/basic conditions. Sapphire—the strongest chemical form of alumina—was partly damaged in some corrosion tests, such as in the acid/alkali boil experiments. Figure 2 (bottom row) shows the SEM images of DLC-AAO and sapphire after boiling in NaOH.

**Table 1:** Comparison of (bio-)chemical resistance of anodic alumina (AAO), sapphire, diamond-like carbon-anodic alumina (DLC-AAO) and diamond.

Chemical	Time	pH	*T* (°C)	AAO	DLC-AAO	Sapphire	Diamond
Saturated Potassium/Sodium Hydroxide (KOH/NaOH)	24 h	14	25	etched **	resistant **	resistant	resistant
Saturated Potassium/Sodium Hydroxide (KOH/NaOH)	2 h	14	80	etched	resistant	damaged	resistant
Phosphoric Acid (10% vol.)	12 h	4	60	etched	resistant	resistant	resistant
Perchloric acid (HClO_4_ 25% vol.)	1 h	1	25	etched	resistant	resistant	resistant
Hydrofluoric Acid (HF 40% vol.)	72 h	3.5	25	etched	resistant	damaged **	resistant
Sulfuric Acid and Sodium Nitrate (1 mL H_2_SO_4_ + 0.25 mg NaNO_3_)	1 h	1	200	etched	resistant	damaged	resistant
Accelerated aging * (Saline buffer)	6 months *in vivo*	5.5	80	damaged	resistant	resistant	resistant

* Saline chamber for 18 days at 80 °C, equivalent to 6 months *in vivo* life span; ** Resistant: Impervious to the specific chemical during the chemical test (unchanged); Damaged: Structural damage caused to the surface during the chemical test; Etched: Fully dissolved during the chemical test.

On the other hand, the DLC-AAO membrane resisted harsh chemical attacks even at an elevated temperature as high as 200 °C (acid boil experiment). The acid boil treatment is a technique routinely used in the diamond community to clean off any residual impurities and sp^2^-bonded carbon from diamond surface. This result clearly shows that the conformal coating of DLC layer is the key factor for the chemical stability of DLC-AAO membranes. The thin, yet conformal DLC coating, confers the chemical stability of the membrane, resulting a structure which is resistant to variety of chemicals (1 < pH < 14). The excellent corrosion resistivity can also be beneficial for device fabrication process, allowing usage of various chemicals for different purposes, such as functionalization or sterilization.

Plasma treatment has been employed widely for coating or surface cleaning of the biomedical devices, especially for the purposes of sterilization. Therefore, biodevices should ideally be resistant to plasma-assisted dry chemical processes. The stability of the proposed electrodes against dry etching was tested using a plasma reactor. Hydrogen plasma (60 Torr, 760 sccm and 1500 W power) was used to test the resistance of the materials to plasma etching. Figure 2 (top row) shows SEM images of DLC-AAO and sapphire after plasma etching, which clearly suggests that alumina (even in its strongest chemical form) is not as resistive as DLC coated alumina against dry etching.

**Figure 3:**
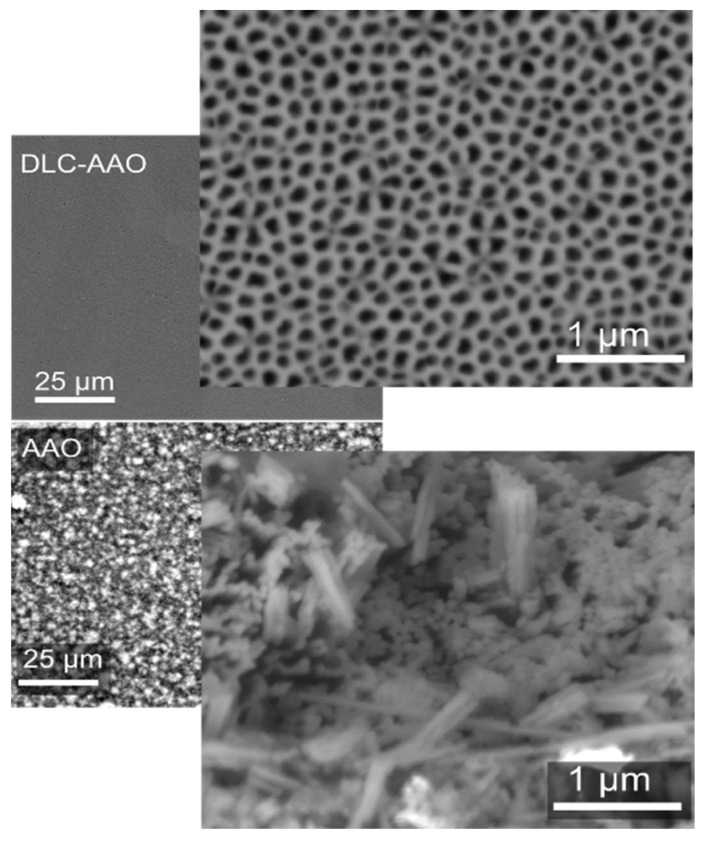
Comparison between the bio-chemical stability of AAO and DLC-AAO electrodes after accelerate aging at 80 °C in medical grade saline solution.

To test the bioresistivity of the fabricated sensors, the samples were soaked in medical grade sterile saline using an environmental test chamber for 18 days at 80 °C. This is equivalent to an *in vivo* life span of six months [41]. SEM micrographs of the samples after the test are shown in Figure 3. While an AAO membrane was partially etched, a DLC-AAO membrane maintained its original structure and exhibited no signs of degradation. This is another piece of evidence that DLC coated AAO provides a highly stable material for long-term *in vivo* applications.

**Figure 4:**
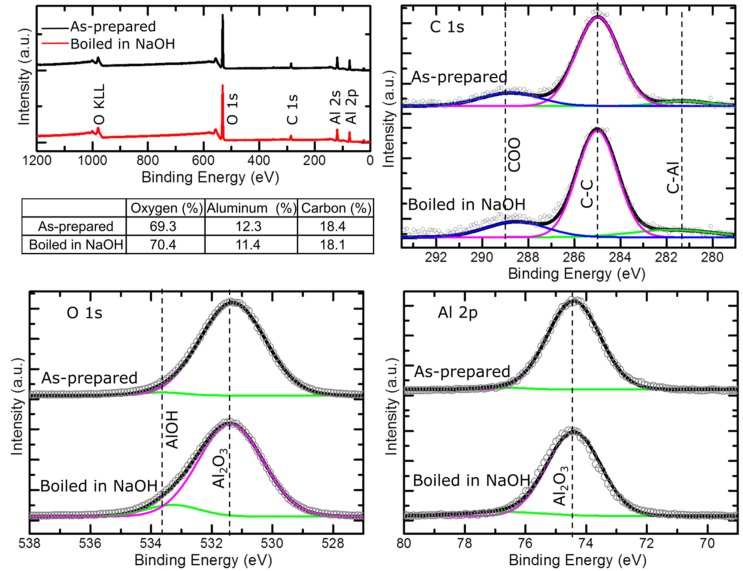
X-ray photoelectron spectroscopy (XPS) of the DLC-AAO membranes before and after cleaning with strong alkali (boiling NaOH). It is observed that the surface chemistry of DLC-AAO membrane did not change by this cleaning step.

The chemical composition of the DLC-AAO was investigated using X-ray Photoelectron Spectroscopy (XPS) after and before chemical corrosion. DLC-AAO membranes were washed with boiling NaOH, a technique to clean the surface of the bio-devices. The surface chemistry of the membrane was compared before and after chemical cleaning using XPS. The chemistry (C 1s, O 1s and Al 2p peaks and the elemental composition) of the surface did not change after chemical attack (Figure 4). This property of the membranes can be very useful for sterilization, recycling or regeneration of the bio-devices. (For an in-depth and detailed surface analysis study on bare AAO and DLC-AAO, please refer to our previous publication [33]).

### 2.3. Cytotoxicity

**Figure 5:**
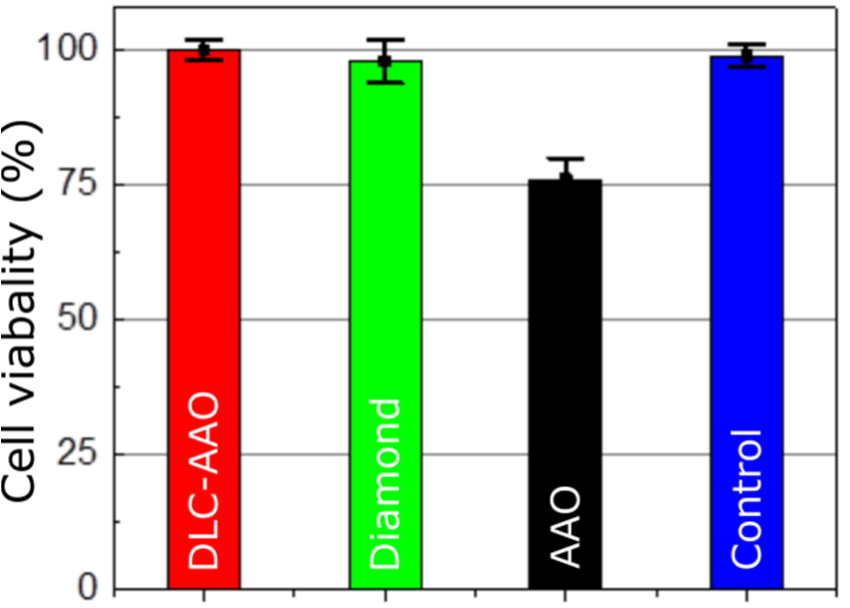
Cytotoxicity of different materials (cytotoxicity test—3T3 fibroblast). DLC-AAO, diamond and the control (tissue culture plastic) are nontoxic, while AAO exhibits moderate toxicity.

Non-toxicity is another crucial factor of a reliable bio-device. The *in vitro* cytotoxicity test is a good measure of biocompatibility because it indicates how a biological tissue/cell will respond to the bio-material. Two standard tests were chosen to study the toxicity of the proposed electrodes. In the first method (non-direct), the electrodes were soaked in Dulbecco’s Modified Eagle Medium (DMEM) and then the extracts were collected and exposed to mouse fibroblasts (3T3 fibroblast). This is a standard test (ISO 10993-12) which mimics or even exaggerates the clinical conditions where the test material is used. According to the results of the experiment, AAO showed moderate toxicity, while diamond and DLC-AAO were clearly non-toxic—similar to the control experiment on silica (Figure 5). The biocompatibility of DLC-AAO is comparable to diamond, which is acknowledged as a biocompatible material and is used in a variety of medical applications, such as bionic devices and orthopedic implants [17,21,42].

**Figure 6:**
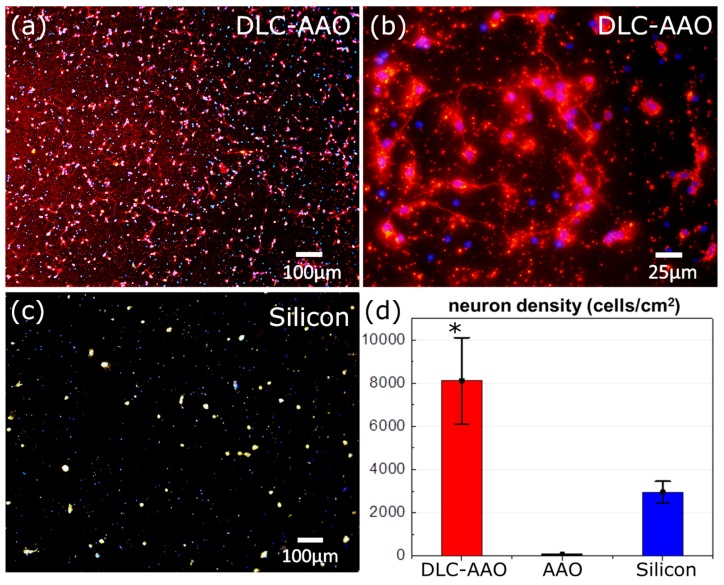
(**a**,**b**) Neuron growth on DLC-AAO, where cell nuclei (blue) and neuronal somata and neurites (red) were stained with DAPI (4',6-diamidino-2-phenylindole) and the neuronal marker βIII-tubulin, respectively. (**c**) Neuron growth on silicon and (**d**) comparison between neuron density on DLC-AAO, AAO and Si. Error bars represent standard deviations of mean values as determined from at least five different area on the samples. * indicate a significant difference from control (tissue culture plastic, TCP) by student’s *t*-test (* *p* < 0.005).

In the second method, the biocompatibility of the electrodes was tested by cultivation of neural cells. Testing the cytotoxicity using primary neurons provides information not only on survival responses but also allows determination of morphological characteristics, such as neurite outgrowth, which is also important in terms of assessment of neural compatibility. In this experiment, electrodes were directly put in contact with neuronal cells and we observed the survival and growth of the neurons after one day. Optical images of the fluorescent neurons on tested electrodes demonstrates that the density of the neurons grown on DLC-AAO electrode was higher than on a silicon/diamond reference, while no cell growth was observed on an AAO sample (Figure 6).

**Figure 7:**
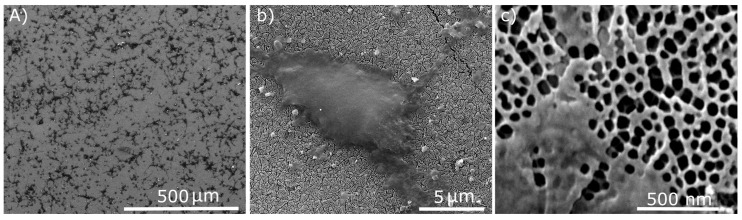
SEM images of neural cortical cells on nanoporous DLC-AAO membranes after one day of cultivation. (**a**) Neurons spread homogenously all over the surface without any aggregation. (**b**) A healthy multipolar cell. (**c**) Early stage of neurite growth on nanopores.

Figure 7 shows SEM images of healthy neurons and neurite outgrowth on the porous DLC-AAO electrodes after one day of cultivation. Figure 7a shows that the neural cortical cells on DLC-AAO were spread homogenously all over the surface without any aggregation or cluster formation. Cells had smooth oval somata, and neurites with uniform shape and diameters. Figure 7b shows that neural cells on DLC-AAO were multipolar (3–4 neurites per cell), which is an enhancement compared to bipolar cells grown on diamond or silicon (images are not shown here). Figure 7c reveals the process of neurite growth on the nanoporous structure, which clearly shows that the cells follow the geometry of the substrate even at nanoscales. Although some of the nanopores of the substrate are partially/completely covered by the cell, we could not confirm the growth of the cells inside the nanopores. The results suggest that the nanoporous structure provides a better neuron interface compared to the flat substrates.

In summary, it is shown that: (i) the biocompatibility of DLC results in promotion of neuron growth in DLC-AAO, and (ii) the nanoporous structure of the DLC-AAO enhances neuron growth when compared with planar surface. These conclusions are in line with other studies where it is shown that the nano-structured scaffolds augment cell-material interactions at different steps, such as initial attachment, differentiation of neuronal lineage, and neuronal phenotype and length [26,27,28,29,30,43,44,45,46,47,48,49,50,51,52,53,54,55,56,57]. It has also been demonstrated by others that nano-topographical features can act as guidance cues to glia and neuron growth on the surface [27,45,47,50,51,53,54]. Despite the efforts on understanding this phenomenon, the cellular mechanism underlying these enhancements still remains elusive [26,44]. A study by Kang *et al.* [44] suggests that the developmental acceleration of neurons occurring on a nano-structure can be due to the biochemical inhibition of filopodia formation (filopodia is an actin protrusion at the leading edge of migrating cells, which functions as a sensor of the local environment and has a mechanical role in protrusion [58]). According to this model, neurons can sense nano-topographies through accelerated filopodial activities, which in turn modulate intracellular cytoskeletal dynamics (just as they do *in vivo*) [44]. Our study can support this model, based on our observation on early stages of neurite growth on a nanoporous material (Figure 6c). It was observed that the cellular protrusion is sensitive to the nano-topography and the neurites start to develop on a nano-topographical pattern.

## 3. Experimental Section

### 3.1. DLC-AAO Fabrication

The nanoporous AAO films used in this study were fabricated from a pure aluminum sheet (99.999%). First, the 1 × 1 cm^2^ aluminum sheets were sonicated in ethanol for 5 min and washed with DI water. The sheets were electrochemically polished in a solution of ethanol/perchloric acid (4:1 v/v) for 3 min at 18 V at room temperature. Polished aluminum sheets were anodized for 20 h in a solution of oxalic acid (0.3 mol/L and 40 V) or phosphoric acid (0.001 mol/L and 150 V) to achieve porous structures with pore diameter of approximately 50 and 150 nm, respectively. Finally, a saturated solution of iodine methanol (at 55 °C) was used to selectively remove aluminum from the backside, leaving a free standing anodic aluminum oxide. The resulted structures were AAO electrodes with 50 or 150 nm pore diameter. The thickness of the final structure is estimated to be ~20 µm.

The surface of the AAO films was then coated by ultrathin (2–5 nm) DLC films using a plasma-enhanced chemical-vapor deposition (PE-CVD) method. The details of the CVD fabrication method have been reported elsewhere [33]. Briefly, the conformal coating of diamond-like carbon was performed in a microwave plasma-enhanced chemical vapor deposition (MW-PECVD) chamber (Cyrannus system from Iplas GmbH, Troisdorf, Germany) with a hydrogen and methane gas mixture (750:10 sccm) at 1500 W microwave power and 70 Torr chamber pressure for 14 min. It has been shown previously that the carbon coating process can be used for modification of AAO membranes with different pores size (20–150 nm) and thickness (up to 100 µm thick membrane) [33].

The DLC-AAO electrodes were then oxidized and cleaned by a 30 s exposure to oxygen plasma with 50 W power followed by 10 min exposure to UV light. This short plasma exposure modifies only the surface atomic termination of the membranes by oxygen, while the original structure of the membrane is maintained [33].

### 3.2. Electrical Conductivity Measurement

A plastic mask was prepared to coat the surface with two gold electrodes using electron beam evaporator. The thickness of the evaporated gold was approximately 30 nm. Each gold electrode was 3 mm × 10 mm in dimensions, and the distance between two gold electrodes was 1 mm. The electrical conductivity of the carbon coated AAO electrodes was measured using two-point electrical measurements.

### 3.3. Chemical Tests

Chemical stability of the samples was tested under different acidic and basic conditions (1 < pH <14). The acidic or basic solutions that were used for resistivity tests include: saturated solutions of sodium hydroxide and potassium hydroxide, 40% hydrofluoric acid in water, phosphoric acid (85%) and 25% perchloric acid in ethanol. The acid boil test was performed in a mixture of sulfuric acid (1 mL) and sodium nitride (0.25 mg) at 200 °C for one hour.

To test the bio-resistivity of the samples, they were soaked in medical grade sterile saline (Aerowash Sterile Sodium Chloride Eyewash Solution) using capped glass vials. Using an environmental test chamber (MicroClimate Benchtop Test Chamber Cincinnati Sub-ZeroManufacturer, Cincinnati, OH, USA) set at 80 °C, samples were kept at temperature for a time period of 18 days. Using the Arrhenius equation, this equates to a lifespan of 6 months *in vivo*.

### 3.4. XPS

Laboratory-based XPS analysis was performed in a Thermo-Fisher K-Alpha apparatus (10^−9^ mbar) using an Mg Kα radiation source (*E* = 1253.6 eV) at a power of 300 W using a 400 μm spot size. Samples were grounded and an electron flood gun was used to compensate for charging during the measurements. Photon energies were chosen to ensure maximum surface sensitivity for high resolution core level scans of C 1s (330 eV), O 1s (550 eV) and Al 2p (150 eV). The binding energy of all spectra was calibrated using the Au 4f 7/2 core level at 84.0 eV. The XPS data analysis was done using a Shirley background subtraction and peak fitting using Gaussian functions*.*

### 3.5. Non-Cytotoxicity Test

Non-cytotoxicity (ISO 10993-12) was done on the samples by immersing them in DMEM (supplemented with 5% fetal bovine serum) for 24 h at 37 °C, 5% CO_2_. Extracts were then collected and stored in 4 °C until used for cell culture. Mouse fibroblasts (3T3 fibroblasts) were seeded in 100 µL of fresh DMEM at 3200 cells/well of 96 well and culture for 24 h to obtain an established cell layer. The DMEM was then removed and replaced by 100 μL of the extracts. The cells were then incubated for another 24 h. At the end, 20 μL of Celltiter Aqueous One solution (Promega) was added to each well of the plate and incubated (37 °C, 5% CO_2_) for 4 h. After 4 h, optical densities (OD) of the wells were measured using a spectrophotometer at 490 nm. Background signal of the wells at 690 nm were measured and subtracted. The values of ODs were proportional to the number of the healthy cells. Reported data are the average of 3 measurements with error indicating the standard deviation.

### 3.6. Neuron Growth

Cytotoxicity and biocompatibility testing with cortical neurons was done using standard tissue culture protocols. Cortical neurons were cultured onto DLC-AAO, AAO, diamond, silicon and a reference cultural dish (silica). Briefly, cerebral cortices from one-day-old rats were isolated to obtain cortical neurons. The cortical tissue was dissociated from the meninges. The dissociation involved protease digestion for 20 min using 10 μg/mL DNase 1 and 250 μg/mL trypsin in a buffer (HEPES). The dissociation reaction terminated by using Trypsin Inhibitor and 10 μg/mL Dnase 1. The solution was centrifuged to collect the cells. Finally, cells were diluted in culture medium which contains neurobasal A with 2% B27 supplement, 2 mM Glutamax, 100 μg/ml penicillin and 100 μg/mL streptomycin. The samples were then seeded with the cells at a concentration of 4 × 10^4^ cells/cm^2^. The cell cultures were incubated at 37 °C in 5% CO_2_. Cells were fixed after 24 h with 4% paraformaldehyde for 20 min and permeabilized with methanol. To stain the neurons, rabbit anti-βIII tubulin was used using standard protocols and cell nuceli were stained with the DNA dye 4′,6-diamidino-2-phenylindole (DAPI). The samples were imaged using fluorescent optical microscopy and scanning electron microscopy.

## 4. Conclusions

Surface modification of AAO with DLC has changed the biochemical and physical properties of the membranes, while the structural properties (pore size and shape) was maintained. The few nanometer-thick, yet uniform and continuous nanocarbon coating conferred chemical stability (1 < pH < 14) and biocompatibility, allowing its direct application in biological conditions, such as bionic devices, orthopedic implants and heart valves. The coated electrodes showed extreme resistance to vigorous dry and wet chemical attacks, which are beneficial in practical device fabrication, functionalization and sterilization steps. Neural compatibility of the electrodes improved significantly after coating with nanocarbon by promoting cell adhesion and growth without using additional biomolecules.

Due to the great promise for neural compatibility, we propose nanocarbon coated nanoporous materials as 3D scaffolds, membranes for cell growth and nerve repair. The detailed study of the applications is beyond the scope of the current work but might be interesting for further experiments.
